# Editorial: Interactions Between Biochemical Pathways Producing Plant Colors and Scents

**DOI:** 10.3389/fpls.2022.955431

**Published:** 2022-06-22

**Authors:** Xiumin Fu, Lourdes Gómez-Gómez, Renata Rivera-Madrid

**Affiliations:** ^1^Guangdong Provincial Key Laboratory of Applied Botany and Key Laboratory of South China Agricultural Plant Molecular Analysis and Genetic Improvement, South China Botanical Garden, Chinese Academy of Sciences, Guangzhou, China; ^2^Departamento de Ciencia y Tecnología Agroforestal y Genética, Instituto Botánico, Facultad de Farmacia, Universidad de Castilla-La Mancha, Albacete, Spain; ^3^Centro de Investigación Científica de Yucatán A.C. (CICY), Mérida, Mexico

**Keywords:** anthocyanins, carotenoids, pigments, secondary metabolism, tissues, volatiles

## Introduction

The pigments and volatiles metabolites in plants fulfill the function of helping with pollination and spreading seeds (Sosenski and Parra-Tabla, 2019). These compounds also confer economic value on some fruits, vegetables, and flowers by giving them the important quality factors of color and aroma. In addition, these compounds are particularly interesting from the point of view of human health due to their properties as antioxidants, anti-inflammatory, or anticancer activities (Ávila-Román et al., 2021). The pigments in plants mainly comprise carotenoids, anthocyanins, chlorophyll, and betaines (Tanaka et al., 2008; Sudhakar et al., 2016). Volatile compounds are always divided into categories of terpenoids, phenylpropanoids/benzenoids, and fatty acid derivatives by their synthesis pathway (Maffei, 2010).

It has been widely reported that the composition or content of pigments and volatile compounds changes during the ripening of fruits and the development of flowers. Previous studies mainly focused on single pathway. Now with the development of omics technologies, many studies began to study the correlation between the changes of a metabolite in particular with respect to those associated with global changes of the other metabolites as a whole by combining metabolome, transcriptome, and genome analysis. The articles in this Research Topic are grouped into two themes as follows:

a) Application of omics technologies in the study of biosynthesis and regulation of pigments.
b) The correlation between pigments, volatile or other compounds.

## Application of Omics Technologies in the Study of Biosynthesis and Regulation of Pigments

Carotenoids are biosynthesized mainly *via* the MEP pathway in plants. Most of the genes in the carotenoid biosynthetic pathway have been identified in plants (Ruiz-Sola and Rodríguez-Concepción, 2012). Carotenoid isomerase (CRTISO) is a key enzyme to catalyze pro-lycopene to lycopene in the carotenoid biosynthetic pathway (Pinheiro et al., 2019). Jiang et al. identified the BoaCRTISO function in Chinese Kale. They found this gene could affect the color of the leaf by regulating the carotenoid and chlorophyll biosynthetic gene transcripts. Apart from the biosynthesis genes in the carotenoid pathway, genes related to the formation of carotenoid sequestration structures and the plastids biogenesis also play an important role in carotenoid metabolism in crops (Lu et al., 2006; Lopez et al., 2008). The lower proportions of esterified xanthophylls were caused by the fewer expression levels of xanthophyll esterase (XES), resulting in the pale-yellow flower of petunia (Kishimoto et al., 2019).

A candidate gene *BrWF3*, homologous to *AtPES2*, was found possibly participate in xanthophyll esterification in Chinese cabbage flower (Yang et al.). The SNP deletion of *BrWF3* in the white petals of Chinese cabbage caused the protein to non-function. Therefore, the authors deduced that the lost function of BrWF3 results in the failure combination of carotenoid and polar lipids in the white petals of Chinese cabbage.

The anthocyanin biosynthesis pathway has been reported since 1980 (Holton and Cornish, 1995), and the functions of many genes in the pathway have been identified in recent years. The metabolic pathways and transcriptional regulation of anthocyanins have been intensively studied in model plants. Numerous species in nature could accumulate anthocyanin and may show different levels or types of metabolites. Based on the recent application of omics technologies, we can better understand their metabolites and their regulatory mechanisms. Anthocyanins contribute to *Lycoris* flowers' color. Wang N. et al. identified a hub gene *LrDFR1* through comparative transcriptome analysis, which possibly plays an important role in the anthocyanin accumulation in the *Lycoris* flower. Their experiment results suggested that *LrDFR1* acts as a positive regulator in anthocyanin biosynthesis. Fu et al. carried out integrated transcriptomics and metabolomics analysis in five *C. japonica* cultivars with different color petals and they identified *CjANS* and *Cj4CL* as key contributors to the diversity of petal color of *C. japonica*. Except for structural genes, the transcription factors, such as MYB, bHLH, WD, and MADS-box, play important roles in the regulation of anthocyanin biosynthesis (Zhang et al., 2014). Zhang et al. identified genes responsible for anthocyanin accumulation in the wild blueberry of Wufanshu (*V. bracteatum* Thunb.) by analysis of transcriptomes, and they identified VbMYBA as the transcriptional activator responsible for anthocyanin accumulation. In addition, they found that VbMYBA could activate strong anthocyanin accumulation in tobacco leaves. Li et al. gave a new sight of anthocyanin biosynthesis in peanuts by interaction joint analysis of multi-omics and miRNA. They suggested that *HCT* and chalcone biosynthesis-related candidate genes (*Ah21440, AhCHS*, and *AhCHI*) were the target genes of *AhmiR2950, AhmiR398, AhmiR50*, and *AhmiR51*.

Xiao et al. found that pigment-related compounds could be used as biomarker metabolites for genetic breeding screening. They compared the metabolomes of flower petals of *Nicotiana tabacum* L. (red) and *Nicotiana rustica* L. (orange) species using UPLC-ESI-MS/MS. A batch of novel metabolic biomarkers including flavonoids (kaempferol-3-o-rut, quercetin-glu, and rutin) and carotenoids (lutein and β-carotene) for these species were identified.

## The Correlation Between Pigments, Volatile or Other Compounds

Carotenoids could be oxidized by enzymatic activities (CCD, LOX) or non-enzymatically to generate apocarotenoids in plants, including crocins and bixin, which contribute to pigment development, and β-ionone, α-ionone, β-cyclocitral, β-damascenone, 6-methyl-5-hepten-2-one, and safranal, among others, that contribute to fragrance. The review of Zheng et al. summarized the underlying mechanism of regulation, metabolism, and genetics of apocarotenoid diversity, particularly focusing on apocarotenoid pigments and volatiles. In addition, they proposed a strategy that combines chromatography-mass spectrometry apocarotenoid profiling with multi-omics (such as QTL mapping, GWAS, and RNA-seq) to find new QTLs for apocarotenoid metabolite traits and identify their underlying genes.

Bixin is the second most economically important natural apocarotenoid. Bixin has an orange-red color and is produced mainly from *Bixa orellana* seeds. The bixin metabolic pathway was proposed as early as 20 years ago by Bouvier et al. (2003), but it has not been deeply studied at the biochemical and molecular levels. Us-Camas et al. identified two new genes *BoCCD1* and *BoCCD4* that participate in the biosynthesis of bixin aldehyde, which is the first product of bixin biosynthesis of *Bixa orellana* by using an *in vivo* (*E. coli*) and *in vitro* approach. They analyzed the generated products by LC-ESI-QTOF-MS/MS.

Xi et al. compared the carotenoids and aroma volatile apocarotenoids profiles of fruits of three different colored apricot cultivars. They found the total carotenoid contents were negatively correlated with the transcripts of *CCD1* and *CCD4* genes, while the aroma volatile apocarotenoid contents (mainly β-ionone) were positively regulated. These results suggested that CCD1 and CCD4 may be the key regulatory points for the profiles of color and aroma in apricot fruits. Therefore, these two genes can potentially be used as the targets for molecular breeding.

β-ionone is also a significant contributor to the fragrance of sweet osmanthus flowers and is mainly determined by the CCD4 enzyme (Han et al., 2019). Yan et al. performed a comprehensive analysis of the MYB-related transcription factor superfamily in sweet osmanthus and provided novel insights into the roles of OfMYB-related genes in sweet osmanthus as regulators of volatile compounds. They found that OfMYB1R114 and OfMYB1R70 members accelerated β-ionone formation. While OfMYB1R201 was involved in decreasing the β-ionone content. The mechanism of how these candidate MYB-related genes involved in the regulation of aroma substances remains to be further studied.

The metabolism of carotenoids has been studied intensively for decades, while the interaction mechanism between carotenoids and norisoprenoids (β-ionone) under abiotic stress remained unclear in many plants. Liu et al. found that carotenoid metabolism in peach fruit was significantly influenced by expression levels of carotenoid pathway genes (*PpPSY, PpLCY-B, PpLCY-E*, P*pCHY-B*, and *PpCCD4*) under UV-B irradiation. They deduced that the increased β-carotene and the decreased volatiles β-ionone were partially caused by the inhibition of *PpCCD4* expression level under the UV-B irradiation. These results suggested that some stress factors could affect the carotenoid metabolism and then influence or change the volatile patterns.

Anthocyanins and a variety of aroma compounds (benzaldehyde, phenylacetaldehyde, and methyl salicylate) are derived from phenylalanine *via* the shikimate pathway. However, studies on the underlying mechanism of the relationship between anthocyanins and phenylalanine-derived volatiles are few. Mei, Wan et al. reported a specific tea (*Camellia sinensis*) variety with purple flowers, which accumulate a high concentration of anthocyanins. Meanwhile, tea flowers contain special volatile benzenoid-phenylpropanoids (BPs), such as 1-phenylethanol (1-PE) and acetophenone (AP). What would happen to the volatile compounds when the flower color mutates naturally from white color to purple color? According to the results, they found the flux to the benzenoid-phenylpropanoids (BPs) was also enhanced along with the anthocyanins accumulation in the flower of the tea plant.

In addition to the volatiles generated from the degradation of certain metabolites in plants, such as carotenoids or phenylalanine-derived compounds, volatiles contributing to the aroma of plants are as well directly synthesized, as the case of monoterpenes (linalool, geraniol, and their derivatives), which are derived mainly through the MEP pathway. Huang et al. identified monoterpenes as the main scent components in *Phalaenopsis bellina* flower, and they found that TPS-b and TPS-e/f enzymes are involved in the monoterpene biosynthesis in the P. *bellina* floral scent. Interestingly, the P. *bellina* flower accumulates anthocyanins, but the main aroma released is not phenylalanine-derived volatiles; while this flower could release terpenoid aroma from the MEP pathway, but could not accumulate carotenoids. Therefore, the regulation of different derived volatile compounds is very complex in plants. Ke et al. identified HcMYB1 could activate the structural gene *HcBSMT2* involved in methyl benzoate biosynthesis, and HcMYB2 can also activate the structural gene *HcTPS5* involved in linalool biosynthesis in *H. coronarium* flowers. These findings have shed light on the regulation of volatile compounds in plants.

The changes in plant color could affect not only volatiles but also other metabolism compounds. Mei, Lin et al. compared metabolites in fresh and fermented tea (*Camellia sinensis*) leaves between “Yinghong 9” (green leaves) and “Huangyu” (mutant yellow leaves) cultivars by using targeted metabolomics. Apart from the significant difference in pigments between these two cultivars, they also found alterations in polyphenols and volatiles. Proanthocyanidins are the main pigment substances in brown cotton, and studies have shown that fiber color is negatively correlated with fiber yield and quality. But the underlying mechanism between proanthocyanidins biosynthesis and metabolism in cotton fiber is unclear. Wang Z. et al. investigated the key structure and regulatory genes in the proanthocyanidins biosynthesis of brown cotton by combing with transcriptome co-expression network and metabolome analysis, and thus established the transcriptional regulatory network of proanthocyanidins biosynthesis and flavonoid metabolism in cotton.

Taken together, the Frontiers Research Topic presented here documents recent advances in pigments and volatiles biology research. In the present volume, the authors use advanced omics technologies to elucidate the biosynthesis and regulation of pigments and volatile and address the interactions between pigments and their related secondary compounds in plants. These results would give insight into the interaction between pigments and volatile and inspire further advances in the study of plant metabolites interaction.

## Author Contributions

All authors contributed equally to the manuscript and approved it for publication.

## Funding

This work was supported by the project from the Young Scientists Fund of the National Natural Science Foundation of China (Grant No. 31902074), the Youth Innovation Promotion Association of Chinese Academy of Sciences (2022351), and the CAS Scholarship. RR-M work was supported by the Consejo Nacional de Ciencia y Tecnología (CONACYT) (Fronteras de la Ciencia No. 2016-01-1716).

## Conflict of Interest

The authors declare that the research was conducted in the absence of any commercial or financial relationships that could be construed as a potential conflict of interest.

## Publisher's Note

All claims expressed in this article are solely those of the authors and do not necessarily represent those of their affiliated organizations, or those of the publisher, the editors and the reviewers. Any product that may be evaluated in this article, or claim that may be made by its manufacturer, is not guaranteed or endorsed by the publisher.
